# Changes Occurring in the Size of the Pupil in Cases of “Shell-shock”

**Published:** 1919

**Authors:** 


### OPHTHALMOLOGY AND OTO-LARYNGOLOGY

---

**Changes Occurring in the Size of the Pupil in Cases of " Shell-shock ".** By M. Lacroix. *Bulletin de la Société de Chirurgie de Paris,* May 28, 1918.

19 cases of traumatic shock have been observed by M. Lacroix. He relates : 1) that slight shock does not affect the pupils; 2) that in all severe cases the following facts may be observed :

*a*) A constant immobility of the pupil, whatever quantity of light falls upon it. *b*) The pupil is contracted.

Myosis is rare and exists only in very severe cases. A slight contraction is the rule. When a strong hemorrhage occurs during the shock, the contraction lessens and the pupil has nearly its normal size. It cannot be considered, therefore, as a means of diagnosis of hemorrhage. When hemorrhage is the only cause of the shock, the pupil appears widened.

Just before death, the pupil will widen in all cases.

These disturbances in the pupil's size show that the nervous system, and especially the bulb and sympathetic system, are injured during the shock.

